# High unacylated ghrelin levels support the concept of anorexia in infants with prader-willi syndrome

**DOI:** 10.1186/s13023-016-0440-0

**Published:** 2016-05-04

**Authors:** Veronique Beauloye, Gwenaelle Diene, Renske Kuppens, Francis Zech, Coralie Winandy, Catherine Molinas, Sandy Faye, Isabelle Kieffer, Dominique Beckers, Ricard Nergårdh, Berthold Hauffa, Christine Derycke, Patrick Delhanty, Anita Hokken-Koelega, Maithé Tauber

**Affiliations:** Unité d’Endocrinologie pédiatrique, Cliniques Universitaires Saint-Luc, Université catholique de Louvain, Avenue Hippocrate 10/1300, B-1200 Brussels, Belgium; Unité d’Endocrinologie, Obésité, Maladies Osseuses, Génétique et Gynécologie Médicale. Centre de Référence du Syndrome de Prader-Willi, Hôpital des Enfants, Toulouse, France; Dutch Growth Research Foundation, Rotterdam, The Netherlands; Department of Pediatrics, Subdivision of Endocrinology, Erasmus University Medical Center-Sophia Children’s Hospital, Rotterdam, The Netherlands; IREC, Université Catholique de Louvain, Brussels, Belgium; Axe Pédiatrique du CIC 9302/INSERM. Hôpital des Enfants, Toulouse, France; INSERM U1043, Centre de Physiopathologie de Toulouse Purpan, Université Paul Sabatier, Toulouse, France; Unité d’Endocrinologie Pédiatrique, CHU Dinant Godinne, Yvoir, Belgium; Karolinska University Hospital, Karolinska Institutet, Solna, Sweden; Department of Endocrinology, University Children’s Hospital, Essen, Germany; BESPEED (Belgian Society for Pediatric Endocrinology and Diabetology), Brussels, Belgium; Department of Internal Medicine, Erasmus University Medical Center, Rotterdam, The Netherlands; Unité d’Endocrinologie, Hôpital des Enfants, 330, Avenue de Grande Bretagne, TSA 70034, 31059 Toulouse Cedex 9, France

**Keywords:** Prader-Willi syndrome, Ghrelin, Anorexia, Nutrition, Obesity, Infants

## Abstract

**Background:**

Prader-Willi syndrome (PWS) is a rare genetic neurodevelopmental disorder with different nutritional phases from suckling deficit with failure to thrive to early onset of obesity. Hyperghrelinemia has been described in PWS long before the development of obesity. Ghrelin is found in both acylated (AG) and unacylated (UAG) forms in the circulation. In contrast to AG, UAG has been shown to inhibit food intake and to be elevated in anorexia nervosa. The present project is aiming to determine the underlying mechanisms driving the different nutritional phases in PWS.

**Methods:**

Measurement of at least 4 h-fasting plasma acylated and unacylated ghrelin in 37 infants with a genetic diagnosis of PWS aged from 1 month to 4 years and in 100 age-matched controls without endocrine disorder recruited prior to minor surgery. One blood sampling was analysed for each patient/control and clinical data were recorded. Eleven PWS infants underwent repetitive blood samples at 3 or 6-month intervals during routine visits.

**Results:**

In infants with PWS, AG is not elevated (*p* = 0.45), UAG is significantly higher (*p* = 0.0044; confidence interval 1.06;1.33) resulting in a low AG/UAG ratio (*p* = 0.0056; confidence interval 0.76;0.95) compared to controls.

**Conclusion:**

Unlike children and adults with PWS that have high AG and AG/UAG ratio, infants with PWS have elevated UAG that supports the concept of anorexia in the early phases of the disease. The change in AG/UAG ratio possibly drives the switch from failure to thrive to obesity.

**Clinical trial registration:**

NCT02529085.

**Electronic supplementary material:**

The online version of this article (doi:10.1186/s13023-016-0440-0) contains supplementary material, which is available to authorized users.

## Background

Prader-Willi syndrome (PWS) is a rare genetic neurodevelopmental disorder arising from the lack of expression of paternally imprinted genes in the 15q11-q12 chromosomal region. This syndrome is characterized by various nutritional phases, from suckling deficit with failure to thrive in infancy to early onset of obesity with hyperphagia [1]. The mechanisms driven those different phases are not yet unravelled. In addition to enhance growth hormone secretion, ghrelin has been shown to stimulate appetite and increase adiposity. Ghrelin is found in both acylated (AG) and unacylated (UAG) forms in the circulation [2]. High AG levels have been described as potential cause of hyperphagia and obesity in PWS children and adults [3, 4]. However, hyperghrelinemia has also been described early in infancy in PWS long before the development of obesity [5, 6]. In fact, UAG represents approximately 90 % of the total ghrelin detected in serum and has been shown to inhibit food intake [7, 8]. Interestingly, high UAG levels have been documented in patients with restrictive anorexia nervosa [9–12]. The present project is part of a European study aiming to determine the underlying mechanisms of different nutritional phases in PWS.

We demonstrate normal circulating AG and increased UAG levels in PWS infants compared to age-matched controls thus driving a low AG/UAG ratio, independently from their BMI. This finding supports the concept of anorexia in the early phases of the disease and may drive the switch from failure to thrive to obesity.

## Methods

The study population comprises 37 PWS and 100 control infants with ages from 1 to 48 months. The PWS were followed by two PWS reference centres, in Toulouse, France, and Brussels, Belgium. PWS was genetically confirmed in all patients. The median age at genetic diagnosis was 1 month [95 % confidence interval.0.5–2.5]. Twenty-two (24 %) had a deletion of the 15q11-12 region, 51 % a maternal unidisomy, 3 % a chromosomal translocation, 11 % an imprinting defect, and 11 % lacked a complete genetic study and had abnormal methylation profiles. Sixty-seven percent of the PWS infants started GH treatment at a median age of 13 months [95 % confidence interval 11.0;15.5]. Eleven PWS infants underwent repetitive blood samples (twice in 9; three times in 2) at 3 or 6-month intervals during routine visits. Control infants were recruited prior to minor surgery and their medical records were checked by the study team to exclude endocrine, metabolic and neurological diseases.

The Flanders reference data [13] were used for height and weight and the Niklasson reference data [14] for birth weight, length and head circumference for the Belgian infants, and the French reference data for height and weight, birth weight, length and head circumference [15, 16] were used for the French infants. BMI was calculated as kg/m^2^ and expressed as z-score, adjusted for age and sex. Cole BMI reference data were used for both Belgian and French patients [17].

The nutritional phases are reported as described by Miller et al. [1] and were used to score the eating behaviour of the PWS infants: phase 1a “Hypotonia with difficulty feeding”, phase 1b “No difficulty feeding and growing appropriately on growth curve”, phase 2a “Weight increasing without an increase in appetite or excessive calories”, phase 2b “Weight increasing with an increase in appetite”, phase 3 “Hyperphagia, rarely feels full”, phase 4 “Appetite no longer insatiable”.

Blood samples were collected in the morning after a minimum 4-h fast in all infants. Collection is a critical step to have reliable measurements of ghrelin. To prevent degradation of the plasma ghrelin levels, blood samples were collected in EDTA tubes maintained at +4 °C containing the anti-protease 4-(2-aminoethyl) benzenesulfonyl fluoride hydrochloride (AEBSF, Sigma-Aldrich Chemicals) at a concentration of 2 mg/ml. Blood was centrifuged at 4 °C, and plasma was quickly frozen on dry ice. Samples were stored at−80 °C and assayed within 3–6 months following collection.

Plasma AG and UAG levels were assessed in duplicate (10–50 μL per well) in one laboratory using two-step double antibody sandwich EIAs, obtained from SPIBio (Bertin Pharma, France; A05306 and A05319, respectively). Assays were performed according to the manufacturer’s instructions. In summary, standards, quality controls and samples were incubated in the plate for 2 h at room temperature without tracer. After a 3× wash, tracer antibody was added and incubated for 2 h at room temperature. Following a 5× wash, Ellman’s reagent was added and incubated for approximately 45 min until satisfactory colour development. Last, absorbance was measured at 405 nm using a VictorX4 plate reader (PerkinElmer, Groningen, Netherlands). Data were analysed using Graphpad Prism 5 (La Jolla, California). A sigmoidal third-order (cubic) polynomial fitting was used to determine concentrations from the calibration curves. This resulted in r2 values >0.99 in the majority of the assays. Intra-assay coefficients of variation (CVs) for AG and UAG were 8.2 and 11.4 % and inter-assay CVs for AG and UAG were 3.9 and 11.0 %. CVs were determined over ten and nine assays for AG and UAG, respectively. Samples had inter-duplicate CVs of <20 % for both AG and UAG. The AG/UAG ratio was computed as AG divided by UAG.

Plasma concentrations of insulin were determined using xMAP technology (Luminex, Austin, TX, USA) with 6-plex kits from Millipore (Ref HMHMAG-34 K, Millipore, Billerica, MA, USA). Assays were performed in duplicate for all standards, samples and internal controls in two separate plates that were loaded the same day. Recovery and intra- and inter- assay CVs were calculated to validate the assay. In France, serum IGF-I levels were measured using the IDS-iSYS automated chemiluminescence assay (Immunodiagnostic Systems, UK). In Belgium, IGF-I levels were measured using a two-step double antibody immunoassay (Liaison XL, DiaSorin).

Data are expressed as median [95 % confidence interval] or percent. AG and UAG levels and AG/UAG ratios were log-transformed (natural logarithm) to obtain a normal distribution. Comparative analyses were conducted using Mann–Whitney or Chi-square tests and correlation analysis using linear generalized estimating equations with a common correlation between samples from the same patients, with *p*-value calculation according to Pan et al. and Chaganty et al. [18, 19]. For Additional file 1: Fig. S1, we used nonlinear regressions by B-splines to draw the curves. Because the curves are compatible with linear regressions, we did not use nonlinear regressions for the statistical analysis. A *p*-value <0.05 was considered as significant.

## Results

PWS infants not surprisingly differed from controls in gestational age, delivery mode, birth weight, and breast feeding (Table 1).

**Table 1:** Description of the population

	PWS (*n* = 37)	Controls (*n* = 100)	*P* value^a^
At birth			
Gestational age (weeks)	37.5 (36.0;39.0)	39.5 (39.0;39.5)	0.008
Caesarean delivery (%)	67 %	28 %	<0.0001
Birth length-z-score	−0.85 (−1.08;–0.60)	−0.29 (−0.56;–0.06)	0.010
Birth weight-z-score	−1.65 (−1.90;–1.31)	−0.26 (−0.50;–0.02)	<0.0001
Breast feeding (%)	11 %^b^	66 %	<0.0001
Duration of breast feeding (days)	92 (45;151)	135 (99;167)	NS
At inclusion			
Age (years)	1.8 (1.4;2.3)	2.0 (1.7;2.2)	NS
Sex ratio (% male)	57 %	49 %	NS
Height-z-score	−0.84 (−1.23;–0.48)	0.53 (0.25;0.80)	<0.0001
Weight-z-score	−1.06 (−1.49;–0.64)	0.06 (−0.20;0.35)	<0.0001
BMI-z-score	−0.86 (−1.31;–0.32)	−0.40 (−0.70;–0.10)	NS
GH treatment (%)	68 %	NA	NA
Age at start of GH treatment (months)	13.0 (11.0;15.5)	NA	NA
GH dose (mcg/kg/d)	0.029 (0.027;0.031)	NA	NA
IGF-I (ng/ml)	98 (61;135)	NA	NA
Insulin (pg/ml)	167 (141;194)	178 (160;196)	NS
PWS features			
Age at genetic diagnosis (months)	1.0 (0.5;2.5)		
Type of genetic diagnosis (%)			
Deletion	24 %		
Maternal disomy	51 %		
Translocation	3 %		
Imprinting defect	11 %		
Abnormal methylation profile	11 %		
Nutritional phase at inclusion			
1a [%−age (months(range))]	43 %−4 (2;23)		
1b [%−age (months(range))]	20 %−29 (11;45)		
2a [%−age (months(range))]	23 %−38 (21;46)		
2b [%−age (months(range))]	14 %−34 (26;37)		

Table 1 gives % or Hodges-Lehmann’s medians and their 95 % confidence interval

*Abbreviations*: *NA* Not applicable, *NS* Not Significant

^a^Differences between PWS *vs.* controls were tested with Mann–Whitney or Chi-square tests

^b^Mother’s milk was given without complete breast feeding to PWS infants

**Fig. 1:**
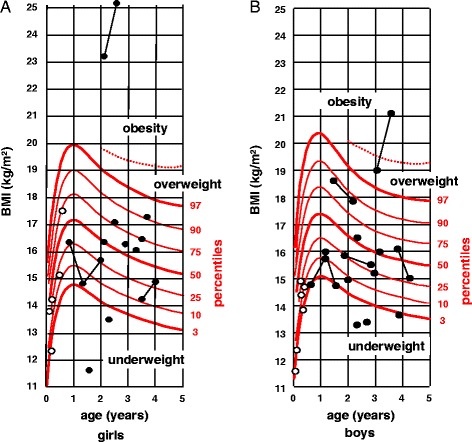
Plotted BMI of the PWS girls **a** and boys **b**. Open circles: PWS infants not treated with GH; dark circles: PWS infants treated with GH. When several measures had been taken for one individual, the circles are linked

These infants were representative of the classical PWS population regarding birth, auxological and anthropometric data (19). PWS infants started growth hormone (GH) treatment at a median age 13.0 months [confidence interval 11.0;15.5]. The age at different nutritional phases is consistent with the description of Miller et al. [1], as shown in Table 1. PWS infants showed normal Body Mass Index (BMI) until 2 years of age, except for six of them, with four being underweight and two obese, as shown in Fig. 1a and b.

**Fig. 2:**
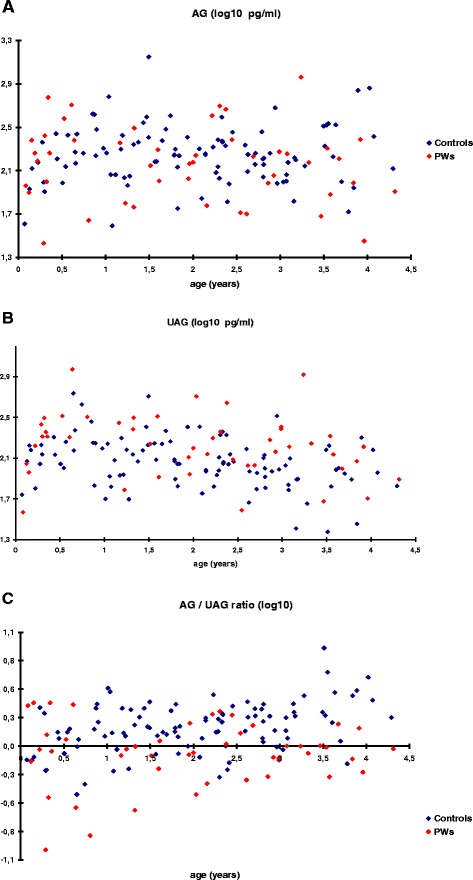
Acylated (AG) **a**, unacylated (UAG) **b** ghrelin levels and AG/UAG ratio **c** according to age in both groups: red: PWS; blue: control children. Comparative analyses were conducted using linear generalized estimating equations with a common correlation between samples from the same patients, with *p*-value calculation according to Pan et al. and Chaganty et al. [18, 19]. PWS *vs.* Controls: *p* = 0.45 (A), *p* = 0.0044 (B), *p* = 0.0056 (C), see Additional file 1: Fig. S1

**Fig. 3:**
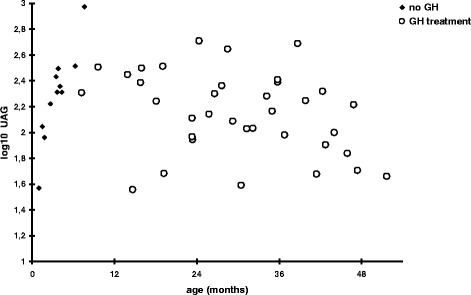
Unacylated (UAG) ghrelin levels according to age in GH-treated (open circles) and untreated (dark squares) PWS infants. Comparative analyses were conducted using linear generalized estimating equations with a common correlation between samples from the same patients, with *p*-value calculation according to Pan et al. and Chaganty et al. [18, 19]. UAG in function of age in GH (−) *vs.* GH (+): *p* = 0.0015

Circulating AG levels were not significantly different between the two groups (*p* = 0.45), whereas higher UAG levels were observed in PWS infants (*p* = 0.0044; ratio = 1.19 [confidence interval: 1.06;1.33] Fig. 2 and Additional file 1: Fig. S1). Consequently, the AG/UAG ratio was significantly lower in PWS infants compared with controls *p* = 0.0056; ratio = 0.85 [confidence interval: 0.76;0.95] Fig. 2 and Additional file 1: Fig. S1). AG levels remained stable between 3 and 48 months in both groups, whereas UAG levels decreased with age (*p* = 0.0031); we do not detect a difference of the slope between PWS infants and controls (*p* = 0.51). Interestingly UAG levels tend to decrease from phase 1a to phase 2b (respectively: median (min-max) 205 pg/ml (37–941) in phase 1a (*n* = 15), 163 pg/ml (36–486) in phase1b (*n* = 13), 175 pg/ml (69–255) in phase 2a (*n* = 8) and 107 pg/ml (39–314) in phase 2b (*n* = 9)) but this is not statistically significant. AG levels were negatively correlated with BMI z-score (*p* = 0.017) and insulin levels (*p* = 0.047), even after adjustment for age and group. UAG levels were negatively correlated with insulin levels (*p* = 0.027), even after adjustment for age and group. No correlation was observed between UAG levels and BMI z-score (*p* = 0.13). AG and UAG levels did not significantly differ between the GH-treated and untreated PWS patients (respectively, *p* = 0.57; *p* = 0.74) (Fig. 3). However, UAG levels are positively correlated with age in the very young infants not yet receiving GH treatment (*N* = 11) (*p* = 0.0088) (Fig. 3). In addition, nasogastric tube feeding did not significantly influence AG and UAG levels (data not shown).

## Discussion

Our results demonstrate for the first time that circulating UAG is elevated in PWS infants and decreases with age, whereas AG is normal and remains stable from 1 to 48 months. Hence, we confirm our previous finding that total hyperghrelinaemia manifests early in life in PWS [5] and we show here that it is due to elevated UAG. High UAG levels with normal AG levels result in a low AG/UAG ratio, particularly in the youngest infants, which may drive the spontaneous poor feeding observed during the first months of life in PWS. Poor feeding that may lead to failure to thrive in the early phases of PWS is classically explained by the presence of severe hypotonia and suckling deficit [20, 21]. Our results suggest that anorexia also plays a role in this poor feeding and may be driven by increased UAG and therefore a relative deficit in AG for their UAG levels. Indeed, UAG has been shown in mice and humans to decrease food intake [7, 8]. Moreover, high circulating UAG and low AG/UAG ratio have consistently been reported in adults with restrictive anorexia nervosa [9–12] in contrast with those with constitutional thinness and normal feeding behaviour who display normal UAG levels [22]. These data suggest that high UAG is more related with feeding behaviour than BMI. Indeed we show in this study that PWS infants have high circulating UAG with low AG/UAG ratio and poor appetite but normal BMI due to adequate calorie intake (data not shown) through nasogastric tube feeding if necessary. We propose that the low AG/UAG ratio reflects anorexia in the postnatal period of PWS. In patients with anorexia nervosa circulating UAG levels normalized after a long-term weight regain. In contrast UAG levels remained elevated in our well-nourished PWS infants suggesting a peculiar dysfunction in this disease and/or at this period of life.

The relative excess of UAG may be due to a defect or abnormal regulation of the unique enzyme that acylates UAG in its serine 3 in mammals, the so-called Ghrelin O-AcylTransferase (GOAT) [23] or, conversely, to increased deacylation of AG by circulating nonspecific deacylases [24]. Although GOAT is a membrane-bound enzyme, it has been shown to circulate in plasma but radioimmunoassay is not yet available, to our knowledge [25]. GOAT is present in human plasma and GOAT protein levels depend on the metabolic environment, with decreased levels in anorexic patients and increased levels in morbidly obese patients [25]. GOAT, as the unique known enzyme acylating ghrelin, may counteract the adaptive changes in ghrelin observed under these conditions and ultimately contribute to the development or maintenance of anorexia and obesity. Studies in rodents and humans suggest that ghrelin acylation can be modified by nutritional status and that the availability of medium-chain fatty acids (MCFAs) is the rate-limiting step for the acylation [26]. Greater insight into the pathophysiological mechanisms is needed. Nevertheless, we have no evidence of an abnormal nutritional status of our PWS infants. Indeed all our PWS infants had adequate calorie intake. Moreover, whatever the BMI (low, normal or increased), it is well known that infants with PWS display an excess of fat mass [27, 28]. Fat mass was measured by DXA scanning in 15 of our PWS patients, AG levels were correlated to fat mass (*p* = 0.000089) but no correlation between fat mas and UAG levels was found (*p* = 0.13, data not shown). Even in the presence of higher fat mass, PWS children are hypoinsulinemic in comparison to age-, gender-matched obese controls [29, 30]. Agreeing with several studies in normal weight and obese children [31] and in PWS children and adults [32, 33], we also showed an inverse relationship between insulin levels and AG and UAG ghrelin levels. As UAG levels are negatively correlated to insulin levels, lower insulin levels could explain high UAG levels. It is however unlikely as we found, at this age, similar insulin levels between controls and PWS infants. GH also did not affect UAG levels. The effect of GH treatment on ghrelin levels has never been described in such young PWS patients. Recently, a study in PWS adults has showed that GH treatment did not affect UAG levels [34].

We recently reported AG and UAG levels in older PWS children and adults in comparison with lean and obese controls [35]. Interestingly, the AG/UAG ratio at this age is increased in PWS due to increased AG and normal UAG levels, which is the opposite situation observed in PWS infants. This suggests an intrinsic defect in AG/UAG in PWS, resulting in a relative AG deficit early in life and a subsequent excess in later life that correlates with the switch in feeding behaviour. The mechanisms remain to be elucidated.

Due to the known effects of AG on brain plasticity, memory and cognition [36] these relatively low AG levels in infants may contribute to PWS patients’ intellectual disability later in life. The early diagnosis of PWS, currently at 1 to 2 months, thus provides a window of opportunity for implementing treatment that may modulate the AG/UAG ratio and thereby may improve both the feeding difficulties and cognition.

## Conclusion

We confirmed that total hyperghrelinaemia is observed at all ages throughout life in PWS, with a different AG/UAG ratio driving opposite phenotypes: anorexia in infants and hyperphagia with deficit of satiety in later life. The mechanism for the switch in this ratio now has to be deciphered. Nevertheless, based on this finding, new therapeutic approaches should focus on the balance of AG/UAG ratio, with drugs to supply either AG or UAG depending on age.

## Ethical approval and consent to participate

The study was approved by the “Comité d’Ethique hospitalo-facultaire des cliniques universitaires Saint-Luc UCL” (reference number BE403201316578) for the centre of Brussels in Belgium and by the “Comité de Protection des Personnes Sud Ouest et Outremer II” (reference number 2-12-25) for the centre of Toulouse in France. Written informed consent was obtained from parents of PWS and control infants.

## Additional file

Additional file 1: Figure S1. Nonlinear regression (± SEM) of acylated (AG) (A), unacylated (UAG) ghrelin (B) levels and AG/UAG ratio (C) according to age in both groups. Black line: control; Red line: PWS infants. For Supplementary Figure1, we used nonlinear regressions by B-splines to draw the curves. Because the curves are compatible with linear regressions, we did not use nonlinear regressions for the statistical analysis. (DOCX 86 kb)

## Abbreviations

AG: acylated ghrelin
BMI: body mass index
GH: growth hormone
GOAT: Ghrelin O-AcylTransferase
MCFAs: medium-chain fatty acids
PWS: Prader-Willi syndrome
UAG: unacylated ghrelin
