# Supplementary material for: High unacylated ghrelin levels support the concept of anorexia in infants with prader-willi syndrome
Source: Orphanet J Rare Dis. 2016 May 4;11:56. doi: 10.1186/s13023-016-0440-0 (PMC4855494; doi:10.1186/s13023-016-0440-0)
Supplement: Additional file 1: Figure S1. — Nonlinear regression (± SEM) of acylated (AG) (A), unacylated (UAG) ghrelin (B) levels and AG/UAG ratio (C) according to age in both groups. Black line: control; Red line: PWS infants. For Supplementary Figure1, we used nonlinear regressions by B-splines to draw the curves. Because the curves are compatible with linear regressions, we did not use nonlinear regressions for the statistical analysis. (DOCX 86 kb) [file 13023_2016_440_MOESM1_ESM.docx]

**Supplementary Figure1**

**Figure S1:** Nonlinear regression (± SEM) of acylated (AG) (A), unacylated (UAG) ghrelin (B) levels and AG/UAG ratio(C) according to age in both groups. Black line: control; Red line: PWS infants. For Supplementary Figure1, we used nonlinear regressions by B-splines to draw the curves. Because the curves are compatible with linear regressions, we did not use nonlinear regressions for the statistical analysis.

**A**

**B**

**C**

UAG (log, pg/ml)

UAG (log, pg/ml)
